# Ischemia Is Related to Tumour Genetics in Uveal Melanoma

**DOI:** 10.3390/cancers11071004

**Published:** 2019-07-18

**Authors:** Niels J. Brouwer, Annemijn P. A. Wierenga, Gülçin Gezgin, Marina Marinkovic, Gregorius P. M. Luyten, Wilma G. M. Kroes, Mieke Versluis, Pieter A. van der Velden, Robert M. Verdijk, Martine J. Jager

**Affiliations:** 1 Department of Ophthalmology, Leiden University Medical Center, Albinusdreef 2, 2333 ZA Leiden, The Netherlands; 2 Department of Clinical Genetics, Leiden University Medical Center, 2333 ZA Leiden, The Netherlands; 3 Department of Pathology, Leiden University Medical Center, 2333 ZA Leiden, The Netherlands; 4 Department of Pathology, Erasmus University Medical Center, 3015 GD Rotterdam, The Netherlands

**Keywords:** uveal melanoma, ischemia, HIF1a, oncology, infiltrate, BAP1, chromosomes, gene expression

## Abstract

Hypoxia-inducible factor 1-alpha (HIF1a) and its regulator von Hippel–Lindau protein (VHL) play an important role in tumour ischemia. Currently, drugs that target HIF1a are being developed to treat malignancies. Although HIF1a is known to be expressed in uveal melanoma (UM), it is as yet unknown which factors, such as tumour size or genetics, determine its expression. Therefore, we aimed to determine which tumour characteristics relate to HIF1a expression in UM. Data from 64 patients who were enucleated for UM were analysed. Messenger RNA (mRNA) expression was determined with the Illumina HT-12 v4 chip. In 54 cases, the status of chromosomes 3 and 8q, and *BRCA1*-associated protein 1 (BAP1) protein expression (immunohistochemistry) were determined. Findings were corroborated using data of 80 patients from the Cancer Genome Atlas (TCGA) study. A significantly increased expression of HIF1a, and a decreased expression of VHL were associated with monosomy 3/loss of BAP1 expression. The relationship between BAP1 loss and HIF1a expression was independent of chromosome 3. The largest basal diameter and tumour thickness showed no relationship with HIF1a. HIF1a expression related to an increased presence of infiltrating T cells and macrophages. From this study, we conclude that HIF1a is strongly related to tumour genetics in UM, especially to loss of BAP1 expression, and less to tumour size. Tumour ischemia is furthermore related to the presence of an inflammatory phenotype.

## 1. Introduction

Uveal melanoma (UM) is the most common intraocular primary malignancy in Caucasian adults, with an incidence of 5.1 per million in the United States (US) [1]. Smaller and medium-sized UMs are often treated with brachytherapy or external radiotherapy (such as electron beam or proton beam irradiation), while larger lesions may require enucleation. Up to 50% of UM patients will develop metastases [2] and, unfortunately, no effective treatment for metastatic disease currently exists. Various prognostic parameters were identified in UM, with a poor prognosis associated with large tumours and ciliary body involvement [3]. Genetic predictors for metastatic tumour behaviour are monosomy 3, gain of chromosome 8q, loss of *BRCA1*-associated protein 1 (BAP1) expression and a Class II gene expression profile [4]. Several new biomarkers were studied, and an increased expression of markers including PRAME [5], ADAM10 [6] and ABCB5 [7] were found to be associated with a greater risk for metastasis in UM.

Recent developments in oncology highlighted the importance of ischemia in the behaviour of solid tumours. Ischemia describes a shortage of oxygen supply from the blood. An important mediator is hypoxia inducible factor 1-alpha (HIF1a), which is increased in ischemic conditions, and activates various pro-angiogenic mechanisms (such as the production of vascular endothelial growth factor (VEGF)) to ultimately regain oxygen [8]. This link between HIF1a and increased vascular growth was established in many malignancies [8,9,10]. The main opponent of HIF1a is the von Hippel–Lindau protein (VHL), which ubiquitinates and degrades HIF1a [11]. VHL is mainly known for its role in the VHL syndrome, a disease caused by a mutation in the *VHL* gene on the short arm of chromosome 3, and this syndrome is associated with the development of various malignant diseases, including hemangioblastoma of the retina and of the central nervous system, and with tumours as renal cell carcinoma (RCC) and pheochromocytoma [12].

In several solid organ malignancies, a high HIF1a expression is related to a poor clinical outcome [8]. This includes colorectal cancer [13] and melanoma of the skin [10]. Mouriaux showed that, in UM, a high HIF1a expression is associated with cell proliferation (MIB-1 expression), expression of the vascular marker CD31 (also known as “platelet and endothelial cell adhesion molecule 1” (PECAM1)) and the cytokine VEGF [14]. Expanding UMs need to attract new blood vessels for their oxygenation, and one would expect that tumour size would, therefore, be a strong determinant of HIF1a expression. While cell culture of UM under hypoxic conditions increased the expression of HIF1a [15], another study reported that HIF1a expression could also be demonstrated in UM cell lines grown under normoxic conditions, implicating that more mechanisms than hypoxia alone are involved in regulating HIF1a [16]. Interestingly, Mouriaux implicated an oxygen-independent mechanism for HIF1a expression, since it was spread throughout the whole tumour and not restricted to hypoxic areas [14]. It is currently not known which tumour factors, such as size, genetics, or the presence of an immune infiltrate, relate to ischemia in UM.

Highly interesting is the development of new drugs that can target HIF1a, as this may provide a new treatment for metastatic UM [17]. More knowledge on the function of HIF1a in UM is needed, however, before this treatment can be fully exploited. We hypothesized that either tumour size or genetic determinants will regulate the expression of HIF1a and set out to investigate which tumour characteristics determine the expression levels of this molecule and its regulator VHL, using a set of patients from the Leiden University Medical Center (LUMC, Leiden, the Netherlands), and subsequently corroborated our findings using data from the Cancer Genome Atlas (TCGA).

## 2. Results

### 2.1. Expression of VHL, but Not HIF1a, Is Related to Clinical Characteristics

Information on tumour mRNA expression was available for 64 UMs. The mean age of the patients was 60.6 years. The median tumour largest basal diameter (LBD) was 13.0 mm, and the median tumour prominence 8.0 mm. The tumour-node-metastasis (TNM) categories of the tumours were T1 in six (9%), T2 in 25 (39%), T3 in 31 (48%), and T4 in two cases (3%). The median follow-up time was 62.0 months (min 2, max 205). In 27 patients (42%), metastases developed, followed by melanoma-related death (Table 1). Nine patients died from other causes.

**Figure 1:**
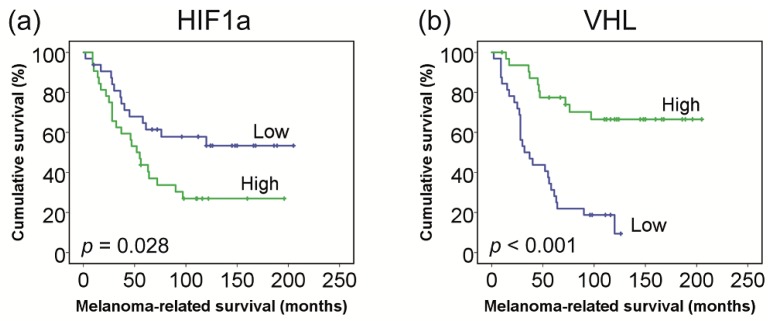
Patient survival in relation to messenger RNA (mRNA) gene expression of (**a**) hypoxia-inducible factor 1-alpha (HIF1a) and (**b**) von Hippel–Lindau (VHL). Groups (high vs. low) were based on the median mRNA gene expression values (*n* = 64).

The median HIF1a messenger RNA (mRNA) expression level in the 64 tumours was 7.21 (range: 6.87–7.95). Expression levels were not associated with gender (*p* = 0.32), age (*p* = 0.11), ciliary body involvement (*p* = 0.40), LBD (*p* = 0.49), tumour prominence (*p* = 0.89), TNM category (*p* = 0.76), or tumour pigmentation (*p* = 0.42). Increased expression was not significantly related to an epithelioid/mixed cell type (*p* = 0.095) and the presence or absence of metastasis (*p* = 0.091) (Table 1). However, a high HIF1a expression was related to worse survival in a Kaplan–Meier analysis (Figure 1).

The median VHL mRNA expression level in the 64 tumours was 7.82 (range: 6.96–8.60). A decreased expression was observed with female gender (*p* = 0.039), older age (*p* = 0.008), ciliary body involvement (*p* = 0.022), larger LBD (*p* = 0.028), the occurrence of metastasis (*p* < 0.001), and worse melanoma-related survival (*p* < 0.001, Figure 1), but was not associated with TNM category (*p* = 0.32), tumour prominence (*p* = 0.85), pigmentation (*p* = 0.29), or cell type (*p* = 0.23) (Table 1).

### 2.2. HIF1a and VHL Expression Are Related to the Presence of T Cells (CD3, CD8) and Macrophages (CD68)

Hypoxia is known to activate various cell types of the immune system, resulting in, e.g., an increased presence of pro-angiogenic macrophages [18]. We wondered how ischemia related to the inflammatory microenvironment in UM, and studied the relationship between HIF1a mRNA gene expression and the presence of various immunological cell types. The expression of HIF1a was related to an increased expression of lymphocyte markers CD3 (*p* < 0.001), CD4 (*p* = 0.027), CD8 (*p* < 0.001), and the macrophage marker CD68 (*p* < 0.001). The expression of VHL was inversely related to infiltrate (CD3: *p* = 0.040, CD8: *p* = 0.012, and CD68: *p* = 0.024) (Table 2).

### 2.3. HIF1a and VHL Expression Are Related to the Expression of Several Angiogenesis-Related Genes

To identify the pathways that are involved in ischemia in UM, we compared the expression of HIF1a and VHL to other genes with relevance to angiogenesis and hypoxia. HIF1a levels showed an inverse correlation with VHL (*p* = 0.002) and VEGF-B (*p* = 0.007), and a significantly positive correlation with PECAM1 (*p* = 0.002) and Von Willebrand Factor (VWF) (*p* = 0.01). While this may point to an increased vascular growth with higher HIF1a expression, no significant correlation was observed between HIF1a and expression of VEGF-A (*p* = 0.68) or endothelial marker CD34 (*p* = 0.34). VHL was related to a significantly increased expression of VEGF-B (*p* = 0.006), and a significantly decreased expression of HIF1a (*p* = 0.002) and PECAM1 (*p* = 0.002) (Table 2).

### 2.4. Tumour Ischemia Is Related to Chromosome 3 and BAP1, but Not to Chromosome 8q

Tumour genetics influence inflammation, as tumours with an extra copy of chromosome 8q are known to have more macrophages, while loss of one chromosome 3 is associated with a complete inflammatory phenotype, containing a mixture of T cells and macrophages [3,19]. We wondered if these genetic aberrations also influence HIF1a levels. We tested this hypothesis in 54 of the 64 enucleated UMs of which chromosome data and information on the expression of the prognostically important BAP1 protein were available. Data on mRNA expression of several genes, including HIF1a and VHL, were also reported in a previous study [20].

Tumours with monosomy 3 (M3) (*n* = 34) had a higher expression of HIF1a (*p* = 0.001) and a lower expression of VHL (*p* < 0.001) compared to tumours with disomy 3 (D3) (*n* = 20) (Table 3). Similarly, BAP1-negative tumours (*n* = 30) had a higher expression of HIF1a (*p* < 0.001) and a lower expression of VHL (*p* = 0.003) compared to BAP1-positive tumours (*n* = 24) (Table 3). This was corroborated using the TCGA data (Table S2).

**Figure 2:**
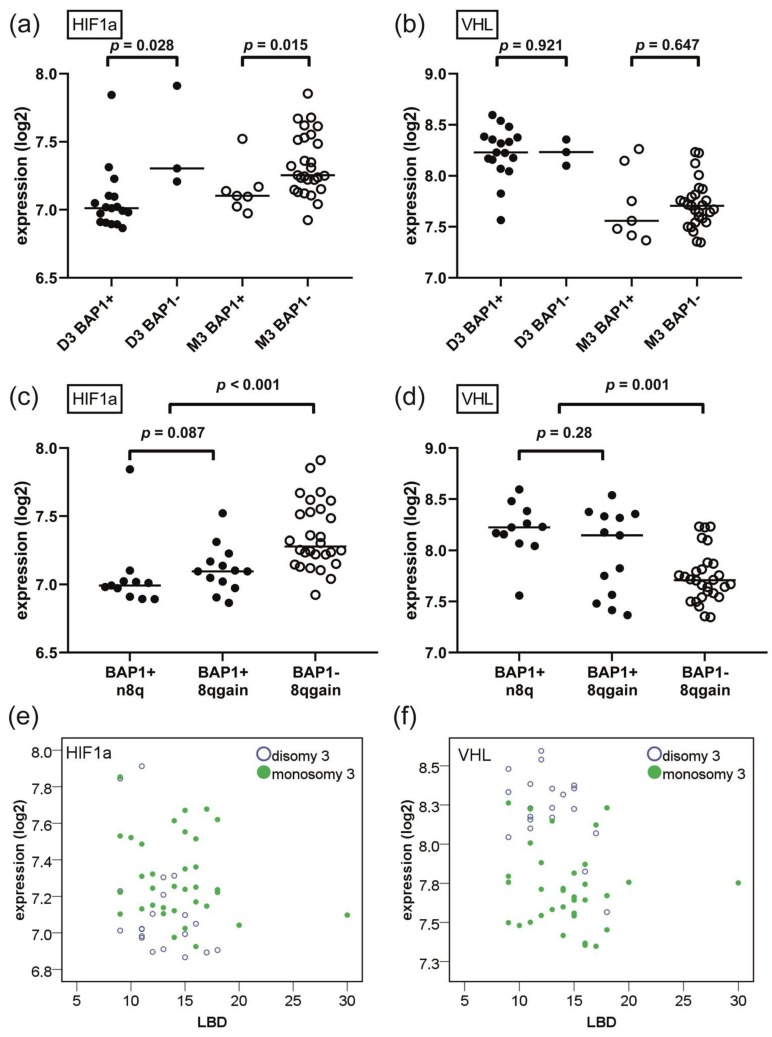
Expression of hypoxia-inducible factor 1-alpha (HIF1a) and von Hippel–Lindau (VHL) mRNA in relation to chromosome status and *BRCA1*-associated protein 1 (BAP1) loss. (**a**) HIF1a expression is significantly increased with BAP1 loss in both disomy 3 (D3) and monosomy 3 (M3) tumours. (**b**) VHL expression is strongly dependent on chromosome 3 status, but is not dependent on BAP1 status within D3 or M3 tumours. (**c**) HIF1a expression is not increased with the early event of 8q gain, but is increased with the later event of BAP1 loss (*n* = 52, excluding two cases of BAP1-n8q). (**d**) As with HIF1a, VHL expression is not dependent on early events as 8q gain, but is decreased with later events as loss of BAP1 (*n* = 52, excluding two cases of BAP1-n8q). (**e**) Adjusted for status of chromosome 3, there is no significant relationship between largest basal diameter (LBD) and expression of HIF1a. (**f**) Adjusted for status of chromosome 3, there is no significant relationship between LBD and expression of VHL. (Abbreviations: BAP1+, BAP1-positive; BAP1−, BAP1-negative; n8q, normal chromosome 8q; 8qgain, gain of chromosome 8q; LBD, largest basal diameter; D3, disomy 3; M3, monosomy 3).

To investigate the role of BAP1 independently of chromosome 3 status, we analysed the association between BAP1 and HIF1a/VHL within the D3 and M3 tumours separately. Within the set of D3 tumours, BAP1-negative tumours (*n* = 3) had a higher expression of HIF1a compared to BAP1-positive tumours (*n* = 17, *p* = 0.028). Similarly, within the set of M3 tumours, BAP1-negative tumours (*n* = 27) had a higher expression of HIF1a compared to BAP1-positive tumours (*n* = 7) (*p* = 0.015) (Table 3). This implies that HIF1a has especially a strong correlation with BAP1 loss, in addition to its correlation with chromosome 3 status (Figure 2a). Interestingly, VHL expression was not related to BAP1 within either the D3 or M3 group (*p* = 0.92 and *p* = 0.65, respectively) (Figure 2b). As the *VHL* gene is located on 3p25, it may not come as a surprise that VHL expression relates more strongly to M3 than to BAP1 loss. In the TCGA data, it was confirmed that BAP1 loss relates to increased HIF1a expression within M3 tumours. No relation could be established within D3 patients, as only one case with BAP1 loss and D3 was present (Table S2).

While loss of BAP1 (or M3) is considered a late event during UM tumour evolution, gain of 8q is considered an early event [21,22]. When analysing all cases, gain of chromosome 8q was associated with an increased expression of HIF1a (*p* = 0.003) and a decreased expression of VHL (*p* = 0.001); however, 8q gain itself was associated with BAP1 loss/M3. Within the group of D3 lesions (and also within lesions with both D3 and BAP1 expression), 8q gain was no longer associated with either HIF1a or VHL (all *p* > 0.10) (Table 3) (Figure 2c,d). In the TCGA data, 8q gain was similarly not associated with HIF1a and VHL within D3 or M3 lesions (Table S2).

### 2.5. Ischemia Is More Related to Tumour Genetics Than to Tumour Size

We demonstrated that both HIF1a and VHL are strongly related to tumour genetics, and wondered how this related to tumour size. HIF1a expression was not related to LBD or tumour prominence in a univariate regression analysis (*p* = 0.21 and *p* = 0.67, respectively). In a multivariate model with the addition of either chromosome 3 or BAP1 status, tumour size also did not relate significantly to HIF1a expression (Table 4, Figure 2e). This was equally seen in the TCGA group (Table S3). From this, we conclude that HIF1a relates more strongly to tumour genetics than to tumour size.

The expression of VHL related negatively to tumour LBD (univariate regression analysis, *p* = 0.034), with larger tumours having lower VHL. In a multivariate model which included LBD and BAP1 staining, the relationship between LBD and VHL remained significant (*p* = 0.034). In a multivariate model which included LBD and chromosome 3, the significance of LBD was lost (*p* = 0.17, Figure 2f), indicating that VHL expression depends highly on chromosome 3 status. Within D3 lesions (*n* = 20), a significant relationship was found between LBD and lower VHL (univariate regression analysis, *p* = 0.033), indicating that tumour size may have an initial role in VHL expression, but is overshadowed by the effect of M3 as soon as this event occurs. In the TCGA data, neither LBD nor tumour prominence were related to VHL expression (also not within D3 or M3 cases), stressing the importance of tumour genetics over tumour size for VHL levels as well (Table S3).

## 3. Discussion

We evaluated how the expression of HIF1a and VHL relates to clinical and genetic factors in UM and established that genetics, but not tumour size, are strongly associated with the level of these markers. M3 and BAP1 loss, but not 8q gain, were related to HIF1a expression, while expression of VHL was mainly determined by the status of chromosome 3. Tumour size only had a role in VHL expression in D3 lesions.

The general theory states that angiogenesis is the result of intra-tumoural hypoxia, which leads to the activation of HIF1a, and the subsequent activation of pro-angiogenic factors such as VEGF. There are several hypoxia-independent mechanisms that lead to the activation of HIF1a, however, as was reported in (cutaneous) melanoma for the phosphoinositide 3-kinsase (PI3K) signalling or presence of reactive oxygen species (ROS) and increased nuclear factor kappa-B (NF-κB) activity [23]. Mouriaux found that HIF1a expression was spread throughout UM tissue, and not limited to hypoxic areas, which implicated a hypoxia-independent activation [14]. Our study indicates that loss of BAP1 expression is highly correlated with HIF1a expression in UM, while M3 is highly correlated with loss of VHL expression. These findings help to understand our recent study that demonstrated that tumour angiogenesis is strongly related to the genetic make-up in UM, with BAP1 loss and M3 being related to an increased microvascular density [20].

The gene coding for VHL is located on chromosome 3p25. From renal cell carcinoma (RCC), it is known that chromosome 3 loss leads to VHL inactivation, and a subsequent increase in HIF1a. This HIF1a increase leads to the highly vascular nature of RCC [24]. In our study, the location of the *VHL* gene also explains the strong correlation between chromosome 3 status and expression of VHL. The location of the *VHL* gene also explains why we detected a correlation with several clinical characteristics, such as the link between a decreased VHL expression and ciliary body involvement, which is known to relate to M3 in UM [3].

We identified that BAP1 loss is strongly associated with the expression of HIF1a, even after adjustment for tumour size or chromosome 3 status. As the gene coding for HIF1a is located on 14q23, it is logical that it is less related to the genetic status of chromosome 3 than VHL. The association between BAP1 loss and HIF1a expression was corroborated in the TCGA data with multivariate regression including BAP1 and chromosome 3, LBD, or tumour prominence (Table S3).

While many reports stress the importance of BAP1 loss for events in UM development and behaviour, the mechanisms are not fully understood. Even so, it is unknown how BAP1 loss can cause an increase of HIF1a. The situation is similar in RCC—a tumour that depends highly on VHL—where BAP1 loss was associated with an increased risk of RCC-related death, although the mechanism is also not yet understood [25]. As BAP1 indirectly suppresses NF-κB via transcription elongation factor A like 7 (TCEAL7) (in human oesophageal squamous cell carcinoma) [26], it can be hypothesized that BAP1 loss leads to an upregulation of HIF1a via the NF-κB cascade. Indeed, it was found that BAP1 loss is associated with an increased NF-κB expression in UM [27]. This might also explain the presence of an inflammatory mixed infiltrate, which we describe as being associated with HIF1a expression.

An explanation for the absent relationship between chromosome 8q status and ischemia in our study may be that gain of 8q is an early event (occurring in smaller, non-hypoxic cases of UM) and that 8q gain is not directly related to other strong activators of HIF1a. Gain of 8q may be too early in the angiogenic switch to have a noticeable effect on ischemia. Gezgin et al. demonstrated that gain of 8q is related to the influx of pro-angiogenic macrophages in UM [28]; however, it may be that this precedes the development of marked ischemia.

We established a relationship between HIF1a expression and the presence of macrophages and lymphocytes in UM. Tumour-associated macrophages are studied more often in cutaneous melanoma, as it is clear that they have a pro-angiogenic function, helping the tumour to overcome a hypoxic environment [18]. Moreover, this is of clinical relevance as the presence of M2 macrophages was related to a worse survival in UM [29]. Earlier, it was noticed that hypoxia leads to the polarization of mainly M2 type macrophages [30], which is the predominant type in UM [29]. Another study found that UM stimulated migration of macrophages independent of growth under normoxic or hypoxic conditions; this points towards a non-oxygenation-dependent generation of the inflammatory environment [31], and is in line with our findings that the genetic status, especially with regard to BAP1, is important in determining HIF1a, as well as the macrophage influx, possibly by the earlier mentioned NF-κB pathway.

The relationship between hypoxia and T cells in tumours is far less understood. Doedens et al. found that HIF1a promoted the function of CD8+ T cells [32]. In our study, we confirm that higher HIF1a expression is related to the presence of CD8+ T cells in UM. An interesting homeostatic mechanism may be suggested, however, as macrophages (activated by HIF1a) may suppress the function of antitumour CD8+ T cells [33,34]. The balance between pro-metastatic macrophages and antitumour CD8+ cells may, therefore, be difficult to predict in ischemic UM.

A strength of our study was that data of two independent datasets were available. A limitation was the small number of patients in some sub groups of our analysis, hampering statistical comparison. Another limitation was that all tumour samples were obtained from enucleated eyes, including mainly larger tumours with a limited variation in tumour size. A third limitation was that we studied mRNA expression and not protein levels. In the TCGA, BAP1 loss was based on the median mRNA expression value, and not on immunohistochemistry (IHC), as was possible with the Leiden data.

Future studies could investigate if blocking the HIF1a pathway has true clinical benefit in UM. We demonstrated that expression of HIF1a and VHL as indicators of ischemia are clearly related to tumour genetics and inflammation in UM, and less to tumour size. This information helps us better understand the difference between metastasizing and non-metastasizing UM. It should be investigated if the HIF1a pathway is involved in the behaviour of UM metastases, to identify if targeting HIF1a is a potential treatment for already disseminated UM.

## 4. Materials and Methods

### 4.1. Patient Selection

Tumour samples were obtained from 64 patients who were treated for a UM by primary enucleation at the Leiden University Medical Center (LUMC, Leiden, the Netherlands) between 1999 and 2008. Clinical data were retrieved from patient medical charts, and complemented with survival data from the Dutch national cancer registry (Registratie Applicatie Nederlandse Kankerregistratie (RANK)). The study was approved by the Biobank Committee of the LUMC (19.061.CBO/uveamelanoomlab-2019-2). The tenets of the Declaration of Helsinki were followed.

### 4.2. Histopathology

Tumour material was snap-frozen using 2-methyl butane. Remaining tumour material was fixed in 4% neutral-buffered formalin for 48 h and embedded in paraffin. Haematoxylin/eosin-stained 4-µm sections were reviewed by an ocular pathologist for confirmation of the diagnosis and evaluated for histologic parameters (Largest basal diameter (LBD), prominence, cell type, pigmentation). The eighth edition of the American Joint Committee on Cancer (AJCC) staging manual was used for tumour classification [35].

### 4.3. Gene Expression

Messenger RNA was isolated from frozen tumour material for gene expression analysis using the RNeasy Mini Kit (Qiagen, Venlo, the Netherlands). The Illumina HT-12 v4 chip was used to determine gene expression levels (Illumina, San Diego, CA, USA). Hypoxia- and angiogenesis-related factors were selected based on the literature regarding angiogenesis in UM (Table S4). Probes for CD3, CD8, and CD68 were previously validated [28].

### 4.4. Chromosome Status and BAP1 Status

The QIAmp DNA Mini Kit was used to isolate DNA for single-nucleotide polymorphism (SNP) analysis (Qiagen, Venlo, The Netherlands). Chromosome 3 status was determined with SNP analysis performed with the Affymetrix 250K_NSP chip and the Affymetrix Cytoscan HD chip (Affymetrix, Santa Clara, CA, USA) [22]. Status of chromosome 8q was identified with dPCR. A threshold of >2.1 was defined as having gain of 8q [22]. BAP1 status was assessed with immunohistochemistry (IHC) as previously described [36], using a mouse monoclonal antibody raised against amino acids 430–729 of human BAP1 (clone sc-28383, 1:50 dilution, Santa Cruz Biotechnology, Dallas, TX, USA). Tumours were scored by an experienced ocular pathologist (R.M.V.) on nuclear staining, and categorized as BAP1-positive or BAP1-negative.

### 4.5. TCGA

Analyses were corroborated using mRNA expression data from 80 UM patients from the TCGA project (http://cancergenome.nih.gov/) [37], with a median follow-up time of 26.0 months (Table S1). In this set, BAP1 expression was provided as mRNA expression levels, and dichotomized at the median into BAP1-positive and BAP1-negative cases [28].

### 4.6. Statistical Analysis

Analyses were performed using SPSS version 23. Data were analysed with the Mann–Whitney *U* test due to non-parametrical distribution (numerical parameters, two groups) or the Jonckheere test for trends (numerical parameters, more than two groups). Correlations were assessed using the Spearman’s rho. Linear regression was performed for univariate and multivariate analyses. Survival was analysed using Kaplan–Meier graphs with log-rank tests. The threshold between “high” and “low” expression of HIF1a and VHL was based on the median expression values. Two-sided tests were reported, and *p*-values <0.05 were considered statistically significant.

## 5. Conclusions

Ischemia—as determined by HIF1a expression—is strongly related to tumour genetics in UM. Expression of HIF1a is increased in tumours with monosomy 3 and BAP1 loss, but not with gain of chromosome 8q. Moreover, the role of tumour genetics in expression of HIF1a is more pronounced than the role of tumour size. These findings help to understand the process of angiogenesis in UM, which is a known risk factor for metastasis development. Also, this implies that the genetic make-up of UM should be considered while investigating the efficacy of HIF1a-targeting drugs.

## Figures and Tables

**Table 1:** Clinical characteristics of the study group (Leiden data, *n* = 64), and messenger RNA (mRNA) expression levels of hypoxia-inducible factor 1-alpha (HIF1a) and von Hippel–Lindau (VHL).

	Total	HIF1a		VHL	
Categorical	Cases (%)	Median	*p*-Value	Median	*p*-Value
**Gender**					
Male	33 (52)	7.20	0.32 ^#^	8.04	0.039 ^#^
Female	31 (48)	7.25		7.70	
**Side**					
Right eye (OD)	30 (47)	7.17	0.57 ^#^	7.84	0.84 ^#^
Left eye (OS)	34 (53)	7.24		7.82	
**TNM cat. (8th)**					
T1	6 (9)	7.14	0.76 *	7.96	0.32 *
T2	25 (39)	7.23		8.01	
T3	31 (48)	7.22		7.76	
T4	2 (3)	7.07		7.75	
**Tumour Pigmentation**					
Light	43 (67)	7.15	0.42 ^#^	8.04	0.29 ^#^
Dark	20 (31)	7.27		7.75	
**Cell Type**					
Spindle	22 (34)	7.14	0.095 ^#^	8.08	0.23 ^#^
Mixed + epithelioid	42 (66)	7.24		7.78	
**Ciliary Body Involvement**					
No	40 (63)	7.17	0.40 ^#^	8.03	0.022 ^#^
Yes	23 (36)	7.24		7.70	
**Metastasis**					
No	27 (42)	7.10	0.091 ^#^	8.18	<0.001 ^#^
Yes	37 (58)	7.24		7.71	
**Melanoma-Related Death**					
No	27 (42)	7.10	0.091 ^#^	8.18	<0.001 ^#^
Yes	37 (58)	7.24		7.71	
		Correlation		Correlation	
**Numerical**	**Total**	**Spearman**	***p*** **-Value**	**Spearman**	***p*** **-Value**
**Age**—Median	61.6	0.201	0.11	−0.331	0.008
**LBD**—Median	13.0	−0.088	0.49	−0.274	0.028
**Prominence**—Median	8.0	0.018	0.89	−0.024	0.85

The *p*-values were calculated with the following: ^#^ Mann–Whitney *U* test, * Jonckheere test for trends. Abbreviations: TNM, tumour node metastasis; LBD, largest basal diameter; Cat, category.

**Table 2:** Messenger RNA (mRNA) expression of ischemic and vascular markers, and immune infiltrate. The correlation between hypoxia-inducible factor 1-alpha (HIF1a), von Hippel–Lindau (VHL) expression and other genes is shown (based on the *n* = 64 lesions with data on HIF1a/VHL expression from the Leiden data).

	HIF1a		VHL	
mRNA	Correlation	*p*-Value	Correlation	*p*-Value
**Ischemic Markers**				
HIF1A	NA	NA	−0.380	0.002 *
VHL	−0.380	0.002 *	NA	NA
**Vascular Markers**				
VEGFA	0.052	0.682	−0.003	0.984
VEGFB	−0.333	0.007 *	0.337	0.006 *
VEGFC	−0.049	0.702	−0.147	0.245
PECAM1	0.381	0.002 *	−0.381	0.002 *
CD34	0.121	0.342	−0.085	0.502
VWF	0.321	0.010 *	0.057	0.653
**Immune Infiltrate**				
CD3D	0.428	<0.001 *	−0.257	0.040 *
CD4	0.276	0.027 *	−0.167	0.188
CD8A	0.452	<0.001 *	−0.314	0.012 *
CD68	0.549	<0.001 *	−0.282	0.024 *
**Other**				
BAP1	−0.330	0.008 *	0.554	<0.001 *

The *p*-values were calculated with Spearman correlation; * indicates significant *p*-values. NA, not applicable.

**Table 3:** Analysis of hypoxia-inducible factor 1-alpha (HIF1a) and von Hippel–Lindau (VHL) mRNA expression in relation to chromosome status and BAP1 (based on the *n* = 54 lesions with data on HIF1a/VHL and chromosome status from the Leiden data).

	Total	HIF1a		VHL	
CATEGORICAL	Cases (%)	Median	*p*-Value	Median	*p*-Value
*All patients*					
**Chromosome 3**					
D3	20 (37)	7.02	0.001 *	8.23	<0.001 *
M3	34 (63)	7.24		7.69	
**Chromosome 8q**					
Normal	13 (24)	7.01	0.003 *	8.22	0.001 *
Gain	41 (76)	7.24		7.74	
**BAP1 IHC**					
Negative	30 (56)	7.28	<0.001 *	7.71	0.003 *
Positive	24 (54)	7.02		8.17	
*Sub-group analysis*					
**Within D3 Lesions**					
D3/BAP1−	3	7.30	0.028 *	8.23	0.921
D3/BAP1+	17	7.01		8.23	
**Within M3 Lesions**					
M3/BAP1−	27	7.25	0.015 *	7.70	0.647
M3/BAP1+	7	7.10		7.56	
**Within D3/BAP1+ Lesions**					
D3/BAP1+/n8q	9	6.98	0.200	8.22	1.000
D3/BAP1+/8qgain	8	7.07		8.32	

The *p*-values were calculated with the Mann–Whitney *U* test; * indicates significant *p*-values. Abbreviations: BAP1+, BAP1-positive; BAP1−, BAP1-negative; n8q, normal chromosome 8q; 8qgain, gain of chromosome 8q; D3, disomy 3; M3, monosomy 3; IHC, immunohistochemistry.

**Table 4:** Univariate and multivariate regression analysis on hypoxia-inducible factor 1-alpha (HIF1a) and von Hippel–Lindau (VHL) mRNA expression (based on the *n* = 54 lesions with data on HIF1a/VHL and chromosome status from the Leiden data).

	HIF1a		VHL	
Variables	B (95% CI)	*p*-Value	B (95% CI)	*p*-Value
**Univariate Regression**				
Chr. 3 (M3 vs. D3)	0.17 (0.03; 0.32)	0.019 *	−0.51 (−0.65; −0.37)	<0.001 *
BAP1 (BAP1− vs. BAP1+)	−0.26 (−0.39; −0.14)	<0.001 *	0.31 (0.13; 0.48)	0.001 *
LBD (per mm increase)	−0.01 (−0.03; 0.01)	0.208	−0.03 (−0.05; −0.002)	0.034 *
**Multivariate Regression**				
*Model 1*				
Chr. 3	0.01 (−0.16; 0.17)	0.952	−0.51 (−0.70; −0.33)	<0.001 *
BAP1	−0.26 (−0.43; −0.10)	0.002 *	−0.01 (−0.18; 0.17)	0.957
*Model 2*				
LBD	−0.02 (−0.03; 0.002)	0.086	−0.03 (−0.05; −0.002)	0.034 *
BAP1	−0.27 (−0.40; −0.15)	<0.001 *	0.30 (0.13; 0.47)	0.001 *
*Model 3*				
LBD	−0.02 (−0.04; 0.00)	0.055	−0.01 (−0.03; 0.006)	0.173
Chr. 3	0.20 (0.06; 0.35)	0.006 *	−0.49 (−0.63; −0.34)	<0.001 *
**Univariate Regression**				
*Within D3* (*n* = 20)				
LBD	−0.05 (−0.10; 0.004)	0.067	−0.04 (−0.08; −0.004)	0.033 *
*Within M3* (*n* = 34)				
LBD	−0.01 (−0.03; 0.008)	0.230	−0.006 (−0.03; 0.02)	0.605

* indicates significant *p*-values. Abbreviations: CI, confidence interval; Chr. 3, chromosome 3; LBD, largest basal diameter; D3, disomy 3; M3, monosomy 3.

## Supplementary Materials

The following are available online at https://www.mdpi.com/2072-6694/11/7/1004/s1: Table S1: Clinical characteristics of the TCGA study group, Table S2: Analysis of HIF1a and VHL mRNA expression in relation to chromosome status and BAP1 (TCGA data), Table S3: Univariate and multivariate regression analysis on HIF1a and VHL expression (TCGA data), Table S4: Overview of hypoxia- and angiogenesis-related genes.
