# Supplementary material for: Ischemia Is Related to Tumour Genetics in Uveal Melanoma
Source: Cancers (Basel). 2019 Jul 18;11(7):1004. doi: 10.3390/cancers11071004 (PMC6678476; doi:10.3390/cancers11071004)
Supplement: Supplementary file 1 [file cancers-11-01004-s001.pdf]

# Supplementary Materials: Ischemia Is Related to Tumour Genetics in Uveal Melanoma

**Niels J. Brouwer, Annemijn P. A. Wierenga, Gülçin Gezgin, Marina Marinkovic, Gregorius P. M. Luyten, Wilma G. M. Kroes, Mieke Versluis, Pieter A. van der Velden, Robert M. Verdijk and Martine J. Jager**

**Table S1.** Clinical characteristics of the TCGA study group ($n = 80$).

| | **Total** | **HIF1a** | | **VHL** | |
|---|---|---|---|---|---|
| **CATEGORICAL** | Cases (%) | Median | *p*-Value | Median | *p*-Value |
| **Gender** | | | | | |
| Male | 45 (56) | 9.95 | 0.72 # | 8.05 | 0.95 # |
| Female | 35 (44) | 9.96 | | 7.95 | |
| **Side** | | | | | |
| Right eye (OD) | NA | NA | NA | NA | NA |
| Left eye (OS) | NA | NA | | NA | |
| **TNM cat. (8th)** | | | | | |
| T1 | 0 (0) | NA | 0.71 * | NA | 0.80 * |
| T2 | 14 (18) | 9.85 | | 8.00 | |
| T3 | 32 (40) | 9.91 | | 7.96 | |
| T4 | 34 (43) | 10.00 | | 8.06 | |
| **Tumour pigmentation** | | | | | |
| Light | 39 (49) | 10.30 | 0.012 # | 8.34 | <0.001 # |
| Dark | 41 (51) | 9.75 | | 7.80 | |
| **Cell Type** | | | | | |
| Spindle | 43 (54) | 9.75 | 0.048 # | 7.95 | 0.87 # |
| Mixed + epithelioid | 37 (46) | 10.15 | | 8.07 | |
| **Ciliary body involvement** | | | | | |
| No | 64 (80) | 9.84 | 0.15 # | 7.96 | 0.95 # |
| Yes | 16 (20) | 10.17 | | 8.15 | |
| **Metastasis** | | | | | |
| No | 53 (66) | 9.82 | 0.21 # | 7.95 | 0.35 # |
| Yes | 27 (34) | 10.10 | | 8.07 | |
| **Melanoma-Related Death** | | | | | |
| No | 60 (75) | 9.89 | 0.25 # | 8.00 | 0.12 # |
| Yes | 20 (25) | 10.10 | | 8.01 | |

| **NUMERICAL** | **Total** | Correlation **Spearman** | *p*-Value | Correlation **Spearman** | *p*-Value |
|---|---|---|---|---|---|
| **Age** – Median | 61.5 | 0.048 | 0.67 | −0.135 | 0.23 |
| **LBD** – Median | 16.0 | −0.048 | 0.67 | 0.026 | 0.82 |
| **Prominence** - Median | 11.0 | −0.062 | 0.59 | −0.030 | 0.80 |

The *p*-values were calculated with the following: # Mann-Whitney *U* test, * Jonckheere test for trend. Abbreviations: TNM, tumor node metastasis; LBD, largest basal diameter; Cat, category.

**Table S2.** Analysis of HIF1a and VHL mRNA expression in relation to chromosome status and BAP1 (based on the $n = 80$ cases from the TCGA data).

| | **Total** | **HIF1a** | | **VHL** | |
|---|---|---|---|---|---|
| **CATEGORICAL** | Cases (%) | Median | *p*-Value | Median | *p*-Value |
| *All patients* | | | | | |
| **Chromosome 3** | | | | | |
| D3 | 37 (46) | 9.78 | 0.018 * | 8.43 | 0.004 * |
| M3 | 37 (46) | 10.19 | | 7.95 | |
| **Chromosome 8q** | | | | | |
| Normal | 21 (26) | 9.92 | 0.88 | 8.43 | 0.047 * |
| Gain | 59 (74) | 9.96 | | 7.95 | |
| **BAP1 status** | | | | | |
| Negative | 40 (50) | 10.27 | 0.002 * | 7.91 | 0.023 * |
| Positive | 40 (50) | 9.69 | | 8.27 | |
| *Sub group analysis* | | | | | |
| **Within D3 lesions** | | | | | |
| D3/BAP1− | 1 | 10.75 | 0.17 | 9.36 | 0.11 |
| D3/BAP1+ | 35 | 9.78 | | 8.43 | |
| **Within M3 lesions** | | | | | |
| M3/BAP1− | 34 | 10.35 | 0.014 * | 7.95 | 0.29 |
| M3/BAP1+ | 3 | 8.69 | | 7.30 | |
| **Within D3/BAP1+ lesions** | | | | | |
| D3/BAP1+/n8q | 16 | 10.06 | 0.088 | 8.58 | 0.37 |
| D3/BAP1+/8qgain | 19 | 9.21 | | 7.96 | |

The *p*-values were calculated with the Mann-Whitney *U* test; * indicates significant *p*-values. BAP1 status was based on mRNA expression data, dichotomized at the median into BAP1-positive and BAP1-negative. Abbreviations: BAP1+, BAP1-positive; BAP1-, BAP1-negative; n8q, normal chromosome 8q; 8qgain, gain of chromosome 8q; D3, disomy 3; M3, monosomy 3; IHC, immunohistochemistry.

**Table S3.** Univariate and multivariate regression analysis on HIF1a and VHL expression (based on the $n = 80$ cases from the TCGA data, with known chromosome 3 status in 74 cases).

| | **HIF1a** | | **VHL** | |
|---|---|---|---|---|
| **Variables** | B (95%CI) | *p*-Value | B (95%CI) | *p*-Value |
| **Univariate regression** | | | | |
| Chr. 3 (M3 vs. D3) | 0.53 (0.13; 0.92) | 0.010 * | −0.47 (−0.80; −0.14) | 0.005 * |
| BAP1 (BAP1− vs. BAP1+) | −0.64 (−1.01; −0.28) | 0.001 * | 0.32 (0.001; 0.64) | 0.049 * |
| LBD (per mm increase) | −0.003 (−0.06; 0.05) | 0.92 | 0.007 (−0.04; 0.05) | 0.74 |
| **Multivariate regression** | | | | |
| *Model 1* | | | | |
| Chr. 3 | −0.55 (−1.39; 0.29) | 0.20 | −1.14 (−1.85; −0.43) | 0.002 * |
| BAP1 | −1.21 (−2.05; −0.36) | 0.006 * | −0.75 (−1.46; −0.04) | 0.039 * |
| *Model 2* | | | | |
| LBD | −0.01 (−0.06; 0.04) | 0.67 | 0.01 (−0.03; 0.05) | 0.61 |
| BAP1 | −0.66 (−1.04; −0.28) | 0.001 * | 0.31 (−0.02; 0.64) | 0.061 |
| *Model 3* | | | | |
| LBD | −0.04 (−0.09; 0.02) | 0.21 | 0.02 (−0.03; 0.06) | 0.48 |
| Chr. 3 | 0.60 (0.18; 1.01) | 0.006 * | −0.48 (−0.83; −0.14) | 0.006 * |
| **Univariate regression** | | | | |
| *Within D3 (n = 37)* | | | | |
| LBD | −0.04 (−0.13; 0.04) | 0.33 | 0.01 (−0.06; 0.08) | 0.74 |
| *Within M3 (n = 37)* | | | | |
| LBD | −0.03 (−0.11; 0.05) | 0.47 | 0.02 (−0.04; 0.09) | 0.47 |

* indicates significant *p*-values. Abbreviations: CI, confidence interval; Chr. 3, chromosome 3; LBD, largest basal diameter; D3, disomy 3; M3, monosomy 3.

**Table S4.** Overview of hypoxia and angiogenesis-related genes.

| mRNA | Name | Locus | EntrezID | Probe name | Probe number | Remarks |
|---|---|---|---|---|---|---|
| **Ischemic markers** | | | | | | |
| HIF1A | Hypoxia Inducible Factor-1alpha | 14q23.2 | 3091 | HIF1A_p1 | ILMN_2379788 | |
| VHL | Von Hippel–Lindau | 3p25.3 | 7428 | VHL_p2 | ILMN_1738579 | |
| **Vascular markers** | | | | | | |
| VEGF-A | Vascular Endothelial Growth Factor-A | 6p21.1 | 7422 | VEGFA_p1 | ILMN_2375879 | |
| VEGF-B | Vascular Endothelial Growth Factor -B | 11q13.1 | 7423 | VEGFB_p3 | ILMN_1722855 | |
| VEGF-C | Vascular Endothelial Growth Factor-C | 4q34.3 | 7424 | VEGFC_p1 | ILMN_1701204 | |
| PECAM1 | Platelet Endothelial Cell Adhesion Molecule 1 | 17q23.3 | 5175 | PECAM1_p1 | ILMN_1689518 | Also known as: CD31 |
| CD34 | Cluster of Differentiation 34 | 1q32.2 | 947 | CD34_p1 | ILMN_2341229 | |
| VWF | Von Willebrand Factor | 12p13.31 | 7450 | VWF_p1 | ILMN_1752755 | |
| **Immune infiltrate** | | | | | | |
| CD3D | Cluster of Differentiation 3D | 11q23.3 | 915 | CD3D_p1 | ILMN_2261416 | Lymphocyte marker |
| CD4 | Cluster of Differentiation 4 | 12p13.31 | 920 | CD4_p1 | ILMN_1727284 | Lymphocyte marker |
| CD8A | Cluster of Differentiation 8A | 2p11.2 | 925 | CD8A_p3 | ILMN_2353732 | Lymphocyte marker |
| CD68 | Cluster of Differentiation 68 | 17p13.1 | 968 | CD68_p1 | ILMN_1714861 | Macrophage marker |
| **Other markers** | | | | | | |
| BAP1 | *BRCA1* Associated Protein1 | 3p21.1 | 8314 | BAP1_p1 | ILMN_1768363 | |

© 2019 by the authors. Licensee MDPI, Basel, Switzerland. This article is an open access article distributed under the terms and conditions of the Creative Commons Attribution (CC BY) license (http://creativecommons.org/licenses/by/4.0/).
